# Physical factor therapies for insomnia: a narrative review of mechanisms, clinical evidence, and phenotype-guided applications

**DOI:** 10.3389/fpsyt.2026.1835178

**Published:** 2026-06-22

**Authors:** Shuhui Li, Qiubai Long, Dongfeng Huang, Yurong Mao

**Affiliations:** 1 Department of Rehabilitation Therapy, Seventh Affiliated Hospital, Sun Yat-sen University, Shenzhen, China; 2 Department of Rehabilitation Medicine, Seventh Affiliated Hospital, Sun Yat-sen University, Shenzhen, China

**Keywords:** autonomic nervous system, circadian rhythms, insomnia, neuromodulation, non-pharmacological treatment, physical factor therapies

## Abstract

Although pharmacological treatments for insomnia remain widely prescribed, their long-term use is constrained by risks of tolerance, dependence, and residual daytime effects. This has prompted growing interest in non-pharmacological interventions that target insomnia’s underlying mechanisms. This narrative review examines five physical factor therapies for insomnia: auditory stimulation, phototherapy, thermotherapy, electrical stimulation, and magnetic stimulation. They are classified according to their primary sites of action into peripheral sensory modulation, central neuromodulation, or systemic autonomic regulation. In each context, research findings about circadian entrainment, thermoregulatory promotion of falling asleep, attenuation of cortical hyperarousal, and restoration of autonomic balance are discussed. Clinical evidence from randomized controlled trials and meta-analyses is critically appraised, with emphasis on therapeutic efficacy, safety, and limitations. Overall, physical factor therapies may constitute promising adjunctive options for selected insomnia phenotypes, but current evidence remains heterogeneous and preliminary for several modalities. Recent advances in portable and home-based technologies support their feasibility, although long-term effectiveness, adherence, and cost-effectiveness remain to be established. Future research should prioritize larger sham-controlled trials, objective sleep and circadian outcomes, standardized protocols, biomarker-guided stratification, and closed-loop intervention systems.

## 1 Introduction

Sleep is a fundamental biological process essential for physiological restoration, cognitive performance, emotional regulation, and metabolic homeostasis (1). Sleep disruptions are recognized as contributing to morbidity and reduced quality of life, increasing the risk of cardiovascular disease, metabolic dysfunction, psychiatric disorders, and all-cause mortality (2, 3). The International Classification of Sleep Disorders (4) classifies sleep disorders broadly as insomnia, sleep-related breathing disorders, central disorders of hypersomnolence, circadian rhythm sleep-wake disorders, parasomnias, or sleep-related movement disorders. Among them, insomnia is the most prevalent and imposes the greatest clinical and societal burden. In the U.S. approximately 10% of adults meet the diagnostic criteria for insomnia, but a substantially larger proportion report insufficient or disturbed sleep (5). Organizations such as the American Academy of Sleep Medicine recommend 7 to 9 hours of sleep per night for adults (6), but many fail to achieve that regularly.

Clinically, insomnia most commonly presents as a chronic condition characterized by persistent difficulties in initiating or maintaining sleep, or early morning awakening. It is typically accompanied by significant daytime impairment. The standard diagnostic criteria require symptoms to occur at least three times per week for a minimum of three months, despite adequate opportunity for sleep. Common daytime consequences include fatigue, mood disturbances, cognitive dysfunction, somatic discomfort, and heightened anxiety (7–11). Estimates of insomnia’s prevalence depend on the diagnostic definitions used and population characteristics, but studies consistently find that up to one-third of adults experience insomnia symptoms, with approximately 10–15% meeting the criteria for insomnia (12, 13). The condition disproportionately affects women and older adults, its prevalence increasing steadily across the lifespan (14–17). In the United States, reported annual prevalence rates for insomnia symptoms range from 35% to 50% (18). In Canada the figure is 18%–20% (19), and in Europe 6.6–9.8% (20). In China, the prevalence of insomnia is estimated at around 15% (21), with marked increases observed during the COVID-19 pandemic (22). Insomnia is strongly associated with adverse cardiometabolic and psychiatric outcomes, including hypertension, cardiovascular disease, metabolic dysfunction, depression, and anxiety (23–25). These associations reflect a bidirectional relationship between insomnia and systemic health (26, 27) and are linked to substantial socioeconomic burden, including increased healthcare utilization, reduced productivity, and diminished quality of life (28, 29).

Cognitive behavioral therapy for insomnia (CBT-I) is today recommended as the first-line treatment for insomnia, with pharmacotherapy reserved for selected or short-term use (30). Although CBT-I demonstrates robust and durable efficacy, its implementation is constrained by limited access to trained providers, variable patient adherence, and scalability challenges (30, 31). Medication, including benzodiazepines and non-benzodiazepine hypnotics, offers short-term symptom relief but carries risks of tolerance and dependence, particularly with long-term use (32, 33). These limitations have intensified interest in alternative and adjunct approaches that safely and directly target the neurophysiological mechanisms of insomnia. In this review, “physical factor therapies” refer to device-based, non-pharmacological physical stimulation interventions that use controlled stimuli, such as sound, light, heat, electrical currents, and magnetic fields, to modulate physiological processes involved in sleep.

This narrative review summarizes the mechanisms, clinical evidence, safety considerations, and potential phenotype-guided applications of auditory stimulation, phototherapy, thermotherapy, electrical stimulation, and magnetic stimulation for insomnia and insomnia-related sleep disturbance. Given the heterogeneity of the interventions, populations, and outcomes, a semi-structured search and evidence classification approach was used. The detailed methodology is provided in **Supplementary File S1**. Evidence was categorized by population type into five groups: (a) diagnosed insomnia disorder, (b) insomnia symptoms, (c) comorbid sleep disturbance, (d) circadian/shift-work populations, and (e) mechanistic or preclinical evidence. We classified the clinical efficacy evidence into four levels: exploratory, emerging, moderate, and high.

## 2 The pathophysiology of insomnia

### 2.1 Hyperarousal

Insomnia is increasingly conceptualized as hyperarousal, involving sustained activation of central and peripheral arousal systems across the 24-hour cycle (34, 35). The heightened arousal persists day and night, reflecting a fundamental imbalance between sleep-promoting and wakefulness-promoting neural systems. Hyperarousal is thus regarded as a final common pathway through which diverse etiological factors converge to produce insomnia (36–39).

A predisposing-precipitating-perpetuating (3P) model (40) is commonly used to describe insomnia. Common predisposing factors include genetic vulnerability, altered circadian regulation and instability of the sleep-wake “flip-flop switch” (41, 42). Precipitating factors are typically acute stressors (43). The perpetuating factors can include maladaptive sleep behavior, conditioned arousal, and/or dysfunctional cognitive-emotional processes that sustain insomnia over time (3, 25, 44).

Neurophysiological evidence supports the hyperarousal model. Electroencephalography (EEG) reveals increased high-frequency activity among individuals with insomnia, particularly in the beta and gamma bands during non-rapid eye movement (NREM) sleep (35, 45, 46). This suggests incomplete deactivation of the cortex, which would impair sleep depth. That leads some investigators to characterize insomnia as a “hybrid” or “mixed state” of sleep and wakefulness (36, 38, 39). Such findings provide a direct mechanistic rationale for interventions aimed at reducing cortical excitability and promoting sleep-specific neural oscillations.

### 2.2 Autonomic imbalance and neuroendocrine dysregulation

In parallel with hyperarousal, insomnia is associated with dysregulation of the autonomic nervous system, most commonly manifesting as sympathetic predominance and diminished parasympathetic tone (47, 48). Physiologically, persons with insomnia often exhibit elevated heart rate, increased metabolism, and a higher core body temperature, all reflecting a failure of autonomic downregulation at night (39, 49, 50). Heart rate variability studies further demonstrate reduced vagal modulation and impaired nighttime recovery, which correlates with both subjective sleep complaints and objective indices of sleep fragmentation (49).

Neuroimaging studies provide converging evidence for structural and functional alterations in the networks regulating arousal and emotion (51). Studies have reported reduced gray matter volume in the orbitofrontal and parietal cortices and smaller hippocampus volumes (52, 53). Broader functional abnormalities involving regions such as the anterior cingulate cortex, thalamus, insula and precuneus have also been reported based on neuroimaging (51). In addition, unstable rapid eye movement (REM) sleep may disrupt synaptic plasticity within limbic circuits, further contributing to emotional dysregulation and sleep fragmentation (44). Continued investigation of these distributed neural networks remains essential for refining mechanistic models of insomnia (44, 54).

Elevated evening and nighttime cortisol levels have consistently been reported in individuals with insomnia (36, 55). That would interfere with sleep initiation and maintenance, and it also provides a plausible explanation for the observed link between insomnia and cardiometabolic and psychiatric disorders (23, 24).

### 2.3 Circadian and thermoregulatory dysfunction

Circadian rhythm misalignment and impaired thermoregulation are additional closely intertwined dimensions of insomnia’s pathophysiology. Getting to sleep normally depends on a reduction in core body temperature, distal vasodilation, and increased heat dissipation. This process is often blunted or delayed in insomnia (50, 56, 57). Prolonged sleep latency and increased wake after sleep onset are the result. In contrast, a highly stable and regular distal skin temperature rhythm, with larger day-night amplitude and fewer random fluctuations, is known to be associated with better sleep (58).

### 2.4 Implications for mechanism-based interventions

Insomnia’s multidimensional dysregulation of cortical arousal, autonomic control, circadian timing, and sensory processing (37, 38, 47, 48, 50) helps to explain the varied responses to single-modality interventions commonly observed. And it highlights the limitations of single-modality therapies. Physical factor therapies target specific pathophysiological components of insomnia. Peripheral sensory stimulation may facilitate sleep initiation and circadian alignment; central neuromodulation can attenuate cortical hyperarousal; system-level interventions may restore autonomic balance. Clarifying the linkages involved thus seems essential for rational selection of a treatment, optimizing its parameters, and developing a personalized treatment plan.

## 3 Overview and classification of physical factor therapies

Physical factor therapies use controlled, non-invasive physical stimuli to modulate the biological systems involved in sleep regulation. They work through sensory processing, neural modulation, or autonomic regulation without medication. As a result, they are generally non-invasive and often well tolerated, but long-term safety and effectiveness require modality-specific evidence before routine use can be recommended.

**Figure 1:**
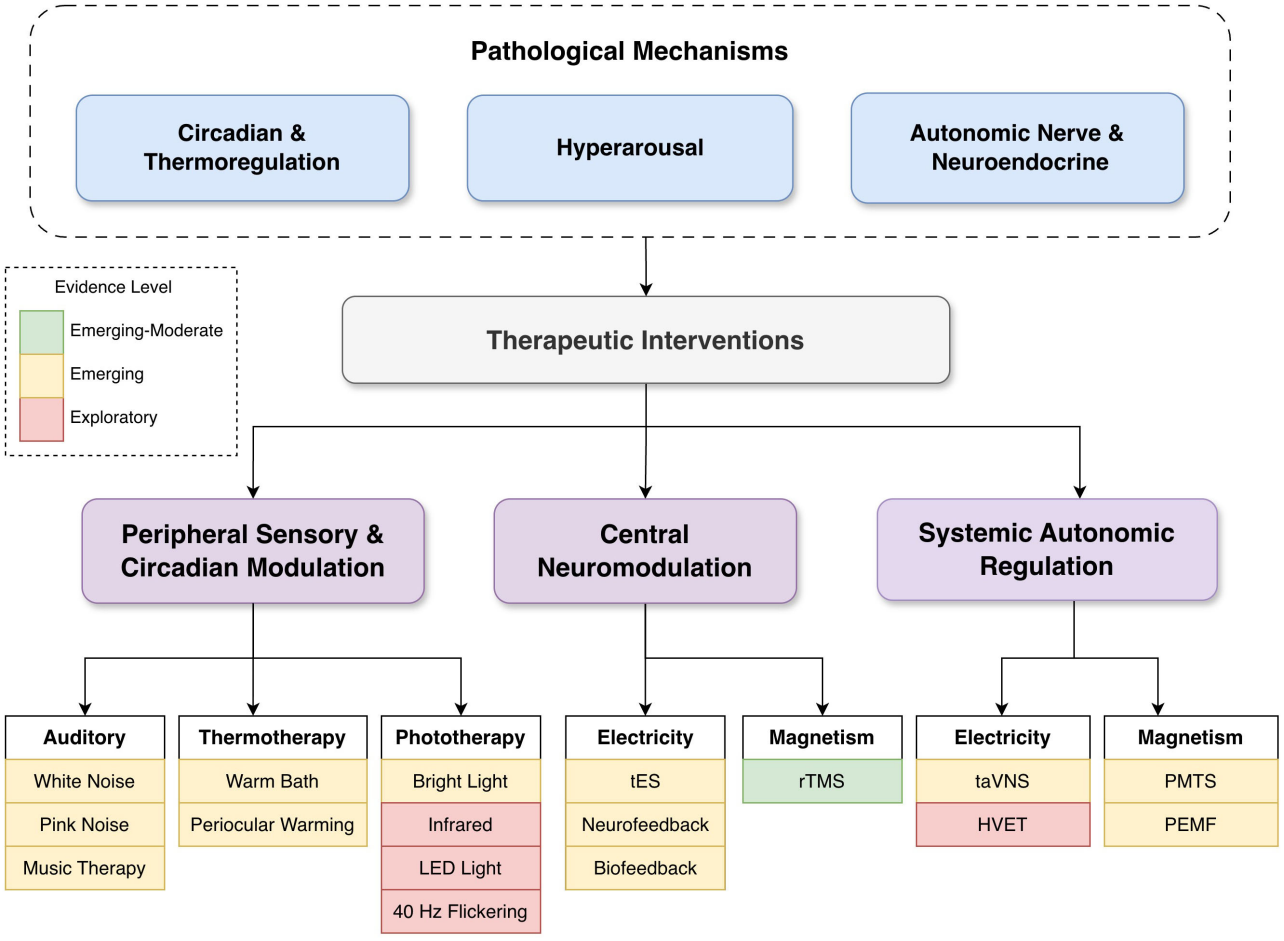
Conceptual framework of mechanism-based physical factor therapies for insomnia. LED, light-emitting diode; tES, transcranial electrical stimulation; rTMS, repetitive transcranial magnetic stimulation; taVNS, transcutaneous auricular vagus nerve stimulation; HVET, high-voltage electrostatic therapy; PMTS, pulse magnetic therapy system; PEMF, pulsed electromagnetic field therapy.

In this review, interventions are classified into three major categories based on their primary site of application, as shown in **Figure 1**. This grouping is organizational rather than mechanistic, as mechanisms often overlap. Thermotherapy is placed under peripheral/circadian modulation because it mainly acts through cutaneous thermoregulatory pathways and heat dissipation, although autonomic effects may contribute. Transcutaneous auricular vagus nerve stimulation (taVNS) is placed under systemic autonomic regulation because it targets vagal afferents, while its downstream effects on the locus coeruleus, raphe nuclei, limbic circuits, and cortical networks overlap with central neuromodulation.

### 1) Peripheral sensory and circadian modulation

This category includes interventions such as auditory stimulation, phototherapy and thermotherapy which act primarily on peripheral sensory systems and circadian regulators. By influencing sensory gating, circadian entrainment, and thermoregulation, these modalities mainly target sleep initiation, sleep timing and early-night sleep consolidation. Their mechanisms are closely aligned with physiological pathways involved in sleep-wake transitions and circadian synchronization.

### 2) Central neuromodulation

The central neuromodulation approaches include transcranial electrical or magnetic stimulation and neurofeedback. They directly target cortical excitability and large-scale neural networks implicated in sleep regulation. These interventions aim to dampen cortical hyperarousal, enhance sleep-promoting oscillations and rebalance arousal-related brain circuits. They are particularly useful for persons experiencing persistent cognitive or cortical arousal.

### 3) Systemic autonomic regulation

The interventions in this category, including vagus nerve stimulation and high-voltage electrostatic therapy, primarily modulate autonomic nervous system activity. By enhancing parasympathetic tone and reducing sympathetic overactivity, these approaches address insomnia’s systemic physiological hyperarousal. Their effects extend beyond sleep itself, potentially influencing cardiometabolic and emotional regulation more generally.

## 4 Auditory stimulation therapies

### 4.1 Mechanism

Auditory stimulation is a peripheral sensory modulation technique. By engaging auditory pathways, it facilitates the transition from wakefulness to sleep through sensory gating and thalamocortical entrainment (59–61). Auditory stimulation is among the sensory-based interventions for sleep modulation most extensively studied. Its therapeutic rationale is grounded in the close coupling between auditory processing, thalamocortical dynamics and sleep oscillations (59). During non-rapid eye movement sleep, slow-wave activity reflects the synchronized firing of cortical neurons which plays a critical role in sleep’s depth and restorative function (59, 62).

Precisely timed auditory stimulation can modulate endogenous sleep oscillations and, when delivered as closed-loop auditory stimulation (CLAS) with controlled timing and intensity, can strengthen slow-wave dynamics without provoking arousal (63–65). Phase-dependent thalamocortical synchronization, and frequency-specific protocols (e.g., 40Hz audiovisual stimulation) have been proposed as mechanisms (62, 63, 66, 67). For instance, such targeted entrainment has demonstrated profound effects on neuroinflammation and memory in Alzheimer’s animal models (66, 67). However, the clinical translation of auditory or audiovisual stimulation specifically for primary insomnia remains highly exploratory.

From a methodological perspective, auditory stimulation approaches for insomnia can be broadly grouped into three paradigms. Open-loop, continuous auditory stimulation with white noise or pink noise primarily creates auditory masking that reduces the salience of environmental noise and stabilizes the sleep environment (68). Phase-targeted CLAS is a more precise approach, directly interacting with endogenous slow oscillations to modulate sleep’s microarchitecture (69–71). Music-based and structured auditory interventions, in contrast, engage affective and limbic circuits, including the amygdala and nucleus accumbens, thereby reducing emotional arousal and facilitating sleep initiation (72–74). Collectively, these paradigms reflect a spectrum ranging from environmental stabilization to direct neurophysiological modulation.

### 4.2 Clinical evidence

Auditory stimulation has shown modality-dependent benefits. White noise and pink noise can improve sleep latency, perceived quality and duration through masking and sensory stabilization (68, 75–77). But if too loud it can trigger arousal, so it is important to respect each individual’s comfort thresholds and noise sensitivity (78, 79).

Phase-locked and CLAS approaches may target sleep microstructure (62–64, 80), but their effects depend on accurate sleep-stage and phase detection and show substantial inter-individual variability. CLAS has been shown to increase slow-wave activity but with notable inter-individual variability (69), while alpha phase-locked stimulation has demonstrated a 29.3% reduction in sleep latency in one trial (70).

Music-based interventions consistently improve subjective sleep metrics, including Insomnia Severity Index (ISI) and Pittsburgh Sleep Quality Index (PSQI), and may also reduce comorbid anxiety and depressive symptoms after multiple weeks of use (72–74). EEG data suggest modulation of functional connectivity relevant to vigilance bias (81). Combining music with cranial electrotherapy stimulation (CES) or real-time EEG-guided alpha modulation may have translational potential for sleep intervention (82, 83). **Table 1** summarizes the clinical evidence and evidence levels for these auditory modalities.

**Table 1:** Auditory stimulation therapies for insomnia.

Therapy	Evaluation item	Evaluation results							
Continuous noise (white/pink noise)	Representative Study Design	Literature	Population	N	RCT	Placebo effect excluded		Objective biomarkers	Follow-up
		(75)	(c) breast cancer patients	104	Yes	No		No	No
		(76)	(c) ICU patients	58	Yes	No		No	Yes
		(77)	(e) healthy adult	12	Yes	No		Yes	No
	Main Clinical Findings & Safety	May improve perceived sleep and reduce noise awakenings. Overall efficacy is mixed, with pink noise showing higher success rates than white noise (68). Safe for short-term use; some report disturbance/fatigue.							
	Limitations	Indirect evidence; small, short studies; limited blinding/placebo control; mostly subjective outcomes; high risk of bias in most studies; and lack of standardized acoustic parameters.							
	Evidence Level	Emerging. Multiple controlled studies suggest potential sleep benefit, supported by mechanistic PSG data, but certainty is limited by indirect populations, lack of placebo/sham control, limited objective clinical outcomes, and inadequate follow-up.							
Closed-loop/phase-locked auditory stimulation (CLAS)	Representative Study Design	Literature	Population	N	RCT	Placebo effect excluded		Objective biomarkers	Follow-up
		(69)	(a) diagnosed by ICSD-3	27	Yes	Yes		Yes	No
		(70)	(b) adults with prolonged sleep onset latency	21	Yes	No		Yes	No
		(80)	(e) healthy adults	10	No	No		Yes	No
	Main Clinical Findings & Safety	Feasible and well tolerated; may shorten objective sleep-onset latency and modulate sleep EEG. Minor skin irritation reported.							
	Limitations	Small samples, short duration, limited blinding/follow-up, mixed subjective benefit.							
	Evidence Level	Emerging. Controlled studies suggest objective benefit and mechanistic plausibility, but evidence is limited by small N, mixed clinical effects, short duration, blinding/placebo concerns, and indirect healthy-volunteer data.							
Music-based intervention	Representative Study Design	Literature	Population	N	RCT		Placebo effect excluded	Objective biomarkers	Follow-up
		(72)	(c) depressed patients	112	Yes		No	Yes	Yes
		(73)	(d) shift-working nurses	360	No		No	No	No
		(74)	(b) college students	75	Yes		No	No	Yes
		(81)	(b) young adults	30	No		No	Yes	No
	Main Clinical Findings & Safety	Overall, music interventions may improve subjective sleep and mood; limited objective EEG/sleep evidence. Generally safe, with no major adverse events reported.							
	Limitations	Heterogeneous populations/interventions; few objective outcomes; no adequate placebo/sham control; limited direct evidence in diagnosed insomnia disorder.							
	Evidence Level	Emerging. Multiple clinical studies suggest potential benefit, including RCT evidence, but certainty is limited by indirect populations, lack of sham/placebo controls, reliance on self-reported sleep, small insomnia-specific samples, short follow-up, and intervention heterogeneity. Current evidence supports music as a low-risk adjunctive option rather than a stand-alone insomnia treatment.							

### 4.3 Summary

Auditory stimulation therapies are generally well tolerated and associated with minimal adverse effects (68). Nevertheless, inappropriate stimulus parameters-particularly excessive intensity or improper phase targeting-may provoke micro-arousals or sleep fragmentation, underscoring the importance of individualized optimization (78, 79). Although the mechanistic rationale is biologically plausible, direct mechanistic validation in insomnia patients remains limited; much of the evidence is inferred from sleep physiology, related populations, or preclinical studies. While auditory stimulation may serve as an adjunctive option for mild to moderate insomnia or sensory hyperreactivity, it appears most suitable specifically for sleep related to environmental noise (68, 77). When persistent cognitive disruption or cortical hyperarousal is involved, auditory interventions alone may be insufficient, and combination with CBT-I or a central neuromodulation approach may be more appropriate.

The findings supporting auditory stimulation in insomnia therapy remain limited by small sample sizes, differing stimulation protocols and inconsistent outcome measures (69, 74, 82). A key limitation is that the same acoustic stimulus may be sleep-promoting for some but arousing for others. And the treatment’s long-term effects and durability have so far been insufficiently characterized. Few studies have systematically examined predictors of response.

Thus, auditory stimulation is non-invasive, accessible and scalable; noise- and music-based approaches may improve subjective sleep experiences and emotional state (72–77), but insomnia-specific efficacy remains emerging. Closed-loop auditory stimulation constitutes a direct sleep modulation technique (69, 80, 83). Future research should prioritize standardizing the technique’s protocols, integrating real-time sleep monitoring, and developing biomarker-guided personalization. At present, auditory stimulation should be considered an emerging, low-risk adjunct, most relevant for noise sensitivity, sensory hyperreactivity, or sleep-onset complaints, rather than a stand-alone treatment for chronic insomnia.

## 5 Thermotherapy

### 5.1 Mechanism

Thermotherapy leverages the body’s natural thermoregulation. By enhancing peripheral vasodilation and heat dissipation, it increases the distal–proximal skin temperature gradient and facilitates the decline in core body temperature that accompanies sleep onset (84–86). Similar device-based thermal interventions are designed to facilitate the onset of sleep (87–90).

In insomnia, this process is frequently disrupted. Core body temperature decline is delayed, there is less distal vasodilation and attenuated circadian amplitude in the skin temperature’s rhythms (50, 56, 57). Impaired thermoregulation thus delays falling asleep. Large-scale community monitoring studies have confirmed that greater diurnal variability and relative amplitude of the distal skin temperature predict better sleep with less waking after falling asleep (58).

Thermotherapy uses peripheral warming to accelerate core body temperature reduction via enhanced heat dissipation (86, 89, 91). Its whole-body approach is applied 1–2 hours before bedtime and it often promotes sleep onset (92, 93).

### 5.2 Clinical evidence

Clinical trials of thermotherapy have frequently reported reductions in sleep onset latency, although most studies remain small, short-term, and heterogeneous. A meta-analysis found that warm foot baths before bed improved subjective sleep quality in older adults, with moderate water temperatures and adequate exposure generating the best outcomes (94). Small randomized trials also support the potential sleep benefits of warm footbath interventions in adults with insomnia symptoms and in older adults (95, 96). While some evidence suggests additional benefit at higher temperatures (94, 95), there are conflicting findings which may reflect individual differences in thermosensitivity. For persons with rheumatoid arthritis, a warm saline or bicarbonate hand or foot bath relieves pain, improving sleep quality (97). Neutral bicarbonate ionized water bathing also showed potential benefits for sleep in adults with stress and sleep dissatisfaction (98). Regular sauna bathing has been associated with better self-reported sleep and well-being, but insomnia-specific evidence is limited (99).

Wearable warming eye masks have shown sleep-promoting effects. Studies in healthy volunteers and individuals with insomnia or sleep difficulty found that periocular warming shortened sleep onset latency and promoted distal heat dissipation (87, 89). Regular bedtime use of a warming eye mask also improved subjective insomnia symptoms and sleep-onset latency in female workers with mild sleep difficulty (90). Other localized temperature-control systems have also shown potential benefits for sleep (88). The thermotherapeutic approaches are summarized in **Table 2**.

**Table 2:** Thermotherapy for insomnia.

Therapy	Evaluation item	Evaluation results							
warm water immersion	Representative Study Design	Literature	Population	N	RCT	Placebo effect excluded	Objective biomarkers	Follow-up
		(88)	(e) healthy adults	11	Yes	No	Yes	No
		(96)	(b) adults	28	Yes	No	Yes	No
		(97)	(c) rheumatoid arthritis patients	54	Yes	No	No	No
		(98)	(b) adults with daily stress and poor sleep quality	25	Yes	No	Yes	No
	Main Clinical Findings & Safety	Warm bath/footbath may improve sleep quality and shorten sleep onset; best around 40–42°C for ≥10 min, 1–2 h before bed (92, 94). Safe and low-cost; avoid burns/falls.						
	Limitations	Small, short studies; varied methods; mostly subjective outcomes; limited blinding/placebo control.						
	Evidence Level	Emerging. Controlled evidence suggests benefit, but certainty is limited by small studies, heterogeneity, risk of bias, and lack of robust large sham-controlled RCTs.						
Periocular warming	Representative Study Design	Literature	Population	N	RCT	Placebo effect excluded	Objective biomarkers	Follow-up
		(87)	(e) healthy male volunteers	18	Yes	Yes	Yes	No
		(89)	(a) diagnosed by DSM-5 (b) individuals with sleep difficulty	38	No	Partly yes	Yes	No
		(90)	(b) female workers	64	Yes	Yes	Yes	No
	Main Clinical Findings & Safety	May improve sleep onset, sleep quality/WASO, and bedtime anxiety; well tolerated with no major safety issues reported.						
	Limitations	Small studies, short duration, limited insomnia-disorder data, no long-term follow-up, some non-PSG measures and possible bias.						
	Evidence Level	Emerging. Sham-controlled evidence suggests benefit, but certainty is limited by sample size, indirect populations, and short follow-up.						

### 5.3 Summary

Thermotherapy appears particularly well suited for older adults, who commonly exhibit age-related impaired thermoregulation and distal vasodilation (56, 57, 91, 94). Individuals with sleep-onset insomnia, heightened stress reactivity, or reduced distal skin temperature at bedtime may be ideal candidates for thermal treatment (56, 89, 90, 96, 98, 100). Japanese-style bathing may facilitate sleep onset, particularly in cold weather, and thermotherapy is generally safe at appropriate temperatures, with caution warranted for individuals with cardiovascular disease, autonomic dysfunction, or impaired thermal sensation (101). The proposed thermoregulatory mechanism is supported by direct observations in insomnia, but clinical benefit should be interpreted primarily from controlled insomnia trials rather than inferred from mechanistic plausibility alone.

Thermotherapy constitutes a relatively cheap, physiologically grounded and clinically accessible intervention for treating insomnia. It reliably promotes falling asleep, and may also improve sleep continuity and depth (86). Nevertheless, the published research has been limited by small sample sizes, diverse intervention protocols, and a predominance of subjective outcome measures (87–90, 95–98). Future research should prioritize standardized yet adaptable thermotherapy protocols, integrating objective thermophysiological and sleep metrics, and identifying biomarkers predicting responses to treatment. At present, thermotherapy is an emerging adjunct most plausibly suited to sleep-onset insomnia associated with impaired heat dissipation, older age, or low distal skin temperature before bedtime.

## 6 Phototherapy

### 6.1 Mechanisms

Retinohypothalamic signaling stabilizes the circadian pacemaker involved in insomnia (41, 102), acting through the suprachiasmatic nucleus (SCN) to regulate sleep–wake timing (42, 103). This suppresses melatonin and modulates hypothalamic–pituitary–adrenal (HPA) linked arousal responses, with bright light increasing cortisol and alertness (104, 105). Importantly, light sensitivity varies substantially between individuals, leading to different circadian responses to the same evening-light exposure (106). There is also preclinical evidence that light may influence sleep through non-circadian mechanisms. Animal experiments suggest that visual flicker stimulation can modulate sleep–wake states through non-circadian mechanisms (107), with 40-Hz flickering light promoting NREM and REM sleep via ENT2- and AMPK-dependent adenosine accumulation in the visual cortex, independent of SCN signaling (108). These findings in rats conceptually expand the scope of phototherapy beyond phase shifting, but their clinical implications remain to be established.

The timing of light exposure appears to influence circadian phases, with morning light associated with phase advances and evening light with phase delays (109). Published studies have used heterogeneous protocols, and therapeutic effects likely depend jointly on timing, dose, duration, wavelength, device distance, and individual light sensitivity. Several studies have used 10,000 lux for about 30min, but no standard protocol can yet be recommended (109). There has also been increasing emphasis on daily light hygiene, including greater daytime light exposure and reduced exposure in the evening and at night to support circadian melatonin secretion (110).

### 6.2 Clinical evidence

The benefits reported for phototherapy include improvements in subjective indices (PSQI or ISI) and objective actigraphy outcomes (109, 111–119). Pooled analyses show that phase-timed delivery is linked to stronger wake after sleep onset (WASO) effects, a pattern directionally supported by insomnia-focused reviews (109, 111). Morning light therapy improved daytime sleepiness in a pilot insomnia randomized controlled trial (RCT) (113). For shift workers, appropriately timed light exposure combined with post-shift light avoidance was found to promote circadian realignment (120). In comorbid depression and insomnia, mechanism studies involving lateral habenula–SCN circuitry have suggested effects on both circadian regulation and mood-related networks (121). Bright light therapy showed potential benefits in clinical sleep-disturbance populations, improving sleep and daytime outcomes in poststroke insomnia and supporting feasible combined chronotherapy after acute coronary syndrome (112, 114). Evidence for red/near-infrared and far-infrared devices remains exploratory, based on small heterogeneous trials with limited objective validation (115–119). Evidence for 40-Hz flickering light is weaker, relying mainly on preclinical/translational findings, with human insomnia data still exploratory (107, 108). An overview of phototherapy is detailed in **Table 3**.

**Table 3:** Phototherapy for insomnia and disturbed circadian rhythm.

Therapy	Evaluation item	Evaluation results							
Bright light therapy	Representative Study Design	Literature	Population	N	RCT	Placebo effect excluded	Objective biomarkers	Follow-up
		(112)	(c) poststroke insomnia patients	56	Yes	Yes	Yes	No
		(113)	(a) diagnosed by DSM-5	14	Yes	No	Yes	No
		(114)	(c) post-acute coronary syndrome patients	15	Yes	No	No	No
	Main Clinical Findings & Safety	Improves subjective sleep quality and insomnia. Modestly increases total sleep time, reduces night awakenings, and slightly boosts sleep efficiency (109, 111, 120). Effects on sleep latency are mixed. Generally safe; side effects are mild and transient (headache, eye strain, glare).						
	Limitations	Small/short trials; inconsistent light parameters (timing/dose/spectrum); limited sham controls and long-term data; mixed objective outcomes.						
	Evidence Level	Emerging. Small controlled studies suggest short-term subjective benefits, but evidence is limited by small samples, indirect populations, limited sham controls, and inconsistent objective outcomes.						
Infrared LED light	Representative Study Design	Literature	Population	N	RCT	Placebo effect excluded	Objective biomarkers	Follow-up
		(115)	(b) adults with subclinical sleep complaints	30	Yes	Yes	Yes	No
		(116)	(d) shift-working nurses	64	Yes	No	No	No
		(117)	(b) older adults	59	Yes	No	Yes	No
		(119)	(e) healthy men	24	Yes	Yes	Yes	No
	Main Clinical Findings & Safety	Small indirect/subclinical RCTs suggest subjective benefits, but objective and insomnia-specific efficacy remains uncertain. Short-term use appears well tolerated.						
	Limitations	Limited by small samples, no long-term follow-up, and most lacking placebo controls. All evaluated indirect populations rather than diagnosed clinical insomnia.						
	Evidence Level	Exploratory. Multiple RCTs suggest subjective benefits and good safety, but the evidence is downgraded due to indirect populations, small sample sizes, absent follow-up, inconsistent objective outcomes, and high risk of bias from inadequate placebo controls.						
40Hz flickering light	Representative Study Design	Literature	Population	N	RCT	Placebo effect excluded	Objective biomarkers	Follow-up
		(107)	(e) sleep-deprived rats	32	No	No	Yes	No
		(108)	(b) children (e) healthy adults (e) mice	49 children, 16 adults, mice	No	No	Yes	No
	Main Clinical Findings & Safety	40Hz light promotes sleep in children with insomnia and alertness in sleep-deprived rats, with no adverse ocular or systemic effects.						
	Limitations	Relies on preclinical models and one uncontrolled pediatric pilot study. Lacks sham-controlled RCTs, long-term follow-up, and direct validation in adult insomnia disorder.						
	Evidence Level	Exploratory. Based only on preclinical data and one uncontrolled pediatric pilot study; lacks adult RCTs and long-term validation.						

### 6.3 Interaction with hyperarousal, blue light, and light hygiene

Wavelength-dependent effects are a critical consideration in phototherapy. Short-wavelength (blue) light around the 460 nm range exerts robust circadian regulation, potently suppressing melatonin secretion (122, 123) and enhancing alertness (124). However, excessive nocturnal exposure to blue light—commonly from electronic devices—can exacerbate insomnia by delaying circadian phasing and sustaining arousal (125, 126). Evening smartphone use before bedtime may attenuate melatonin secretion and influence sleep-related physiology, with blue-light filtering potentially mitigating some effects (125). Conversely, although blue light–blocking glasses are used before bedtime, current evidence shows no significant actigraphic sleep benefits (127).

### 6.4 Summary

Phototherapy is generally safe and well tolerated, with transient eye strain or headache being the most commonly reported adverse effects (109, 111). Caution is warranted for individuals with photosensitive conditions, retinal disease or bipolar disorder, in whom inappropriate light exposure may precipitate adverse responses (109, 111, 128). Crucially, phototherapy’s treatment effects are phase- and timing-dependent; mis-timed light may induce maladaptive circadian shifts and worsen symptoms, depending on the individual’s circadian phase (106, 109, 111). Phototherapy is plausibly positioned for persons with sleep-onset insomnia linked to delayed sleep-wake phasing or circadian instability (109, 129). It is less likely to benefit individuals whose insomnia is dominated by cognitive or cortical hyperarousal.

However, the translation of phototherapy into routine clinical practice faces several notable challenges. Sham controls for bright light are difficult to design (112–114), increasing the risk of placebo and expectancy effects, while optimal parameters such as illuminance, duration, timing, wavelength, and device distance remain poorly standardized (109, 111). Many studies are also small, short-term, and heterogeneous, with limited follow-up (109, 111–119). Evidence for red/near-infrared, far-infrared (115–119), and 40-Hz flickering-light interventions remains preliminary and requires larger controlled trials (107, 108).

In summary, phototherapy can be viewed as a relatively safe and physiologically plausible adjunctive intervention, particularly suitable for insomnia phenotypes associated with circadian misalignment (129). However, mechanistic rationale, especially for infrared and 40-Hz flicker approaches, remains largely preclinical or indirect and should be interpreted as hypothesis-generating rather than proof of clinical efficacy in insomnia. To advance this field, future research would benefit from the development of validated sham-control paradigms and the integration of objective circadian biomarkers (such as dim light melatonin onset) to help guide precise stimulation timing. Overall, bright or scheduled light therapy is an emerging phenotype-specific adjunct for circadian misalignment, whereas infrared and 40-Hz flicker-light approaches remain exploratory.

## 7 Electrical stimulation therapies

### 7.1 Conceptual framework and central electrical neuromodulation

Current evidence tentatively suggests that electrical stimulation therapies may act through central modulation of cortical excitability and sleep-related oscillations and/or peripheral modulation of autonomic function (130–133). Transcranial electrical stimulation (tES) can use either direct current (tDCS) or alternating current (tACS) at low intensity to alter cortical activity. tACS more directly interacts with endogenous neural rhythms (130, 131). Low-frequency stimulation targeting slow brain oscillations has been tested for promoting sleep’s initiation by enhancing sleep-related oscillations. In one randomized study, a wearable 0.75Hz tES device reduced sleep onset latency by more than 53% for individuals with sleep-onset insomnia (130). That frequency approximates that of endogenous slow-wave activity, so these findings are broadly consistent with an entrainment-based explanation, although direct mechanistic evidence in insomnia remains limited. By contrast, approaches such as transcutaneous auricular vagus nerve stimulation are more plausibly linked to autonomic and stress-related physiology than to direct cortical entrainment (132, 134, 135). Interpretation of the published data is complicated by substantial differences in the stimulation parameters tested, in timing, and in the outcome measures reported. Some studies have shown little superiority over their sham conditions (133).

### 7.2 Neurofeedback and self-regulation of cortical arousal

Neurofeedback and biofeedback allow individuals to actively regulate their arousal-related neural activity through operant conditioning. Using real-time feedback about signals such as the beta activity associated with hyperarousal, a person with insomnia can learn to shift their brain activity into more sleep-compatible patterns (136, 137).

Clinical studies have found that neurofeedback may improve subjective sleep quality (136–138) and normalize arousal-related EEG signatures (136, 137). Wang et al. demonstrated that (136) EEG neurofeedback targeting alpha power combined with electromyographic biofeedback can significantly reduce PSQI scores and alter cortical activity in ways consistent with reduced arousal. The evidence remains mixed, however. A meta-analysis (139) has shown that established interventions such as CBT-I often outperform neurofeedback in improving sleep outcomes, raising questions about its utility as a stand-alone therapy. It may be most effective for insomnia subtypes involving pronounced hyperarousal or somatic tension (136, 137, 140).

### 7.3 Peripheral electrical stimulation and systemic autonomic regulation

Peripheral electrical stimulation primarily targets the autonomic nervous system. taVNS is now the technique which has been most extensively studied. taVNS engages nerves projecting to the locus coeruleus, raphe nuclei, and limbic structures to enhance parasympathetic tone and reduce sympathetic dominance (141, 142). An 8-week randomized trial testing taVNS for insomnia showed significantly greater PSQI improvement than the sham control, with the improvement lasting 20 weeks (134). The mechanism may involve vagal afferent signaling and downstream modulation of autonomic and arousal-related brain networks, although direct mechanistic evidence related to insomnia remains limited (132, 142, 143). Pooled safety data indicate mainly mild, transient adverse effects (144).

High-voltage electrostatic therapy (HVET) is an alternative approach. It is hypothesized to modulate cell membrane dynamics and autonomic tone and circulation (145–147). Small, methodologically limited insomnia trials have reported improvements in subjective sleep indices (148–151). However, it is crucial to emphasize that HVET lacks high-quality sham-controlled RCTs and carries a very high risk of bias, largely resting on older, uncontrolled, and small-scale studies. It remains strictly exploratory, and its clinical efficacy cannot be substantiated without rigorous, blinded trials.

### 7.4 Summary

**Table 4** summarizes the diverse electrical stimulation techniques tested. Overall, electrical stimulation therapies offer a mechanistically targeted means of addressing both cortical and autonomic components of insomnia pathophysiology. Central neuromodulatory approaches such as tES and neurofeedback primarily target cortical hyperarousal, whereas peripheral interventions such as taVNS and HVET modulate autonomic balance. All are generally safe, with mild local sensations being the most common adverse effects, but caution is warranted for individuals with implanted electronic devices or a history of seizures (139, 144, 152). tES mechanisms remain only partly demonstrated in insomnia, neurofeedback has some insomnia-specific EEG support but mixed clinical benefit, taVNS mechanisms are largely inferred from autonomic and neurophysiological evidence, and HVET remains mainly speculative.

**Table 4:** Electrical stimulation therapies for insomnia.

Therapy	Evaluation item	Evaluation results							
tES (tACS/tDCS)	Representative Study Design	Literature	Population	N	RCT	Placebo effect excluded	Objective biomarkers	Follow-up
		(130)	(b) adults with sleep-onset insomnia symptoms	24	Yes	Partly yes	Yes	No
		(131)	(a) diagnosed by DSM-5	54	Yes	No	No	Yes
		(174)	(a) diagnosed by ICD-11	157	Yes	No	No	Yes
	Main Clinical Findings & Safety	Mixed but partly positive sleep effects; may improve PSQI, SE, TST, or SOL. Mostly tolerable, but one high-current study raised safety concerns (133).						
	Limitations	Small samples; heterogeneous protocols; limited objective data/follow-up; sham/blinding and reporting issues.						
	Evidence Level	Emerging. Promising but inconsistent evidence; insufficient for routine clinical use.						
taVNS	Representative Study Design	Literature	Population	N	RCT	Placebo effect excluded	Objective biomarkers	Follow-up
		(134)	(a) diagnosed by DSM-5	72	Yes	Yes	No	Yes
		(135)	(a) diagnosed by ICSD-3	40	Yes	Yes	Yes	No
		(143)	(a) diagnosed by DSM-5	67	Yes	Yes	No	No
	Main Clinical Findings & Safety	taVNS improved sleep quality/insomnia severity and some sleep parameters; adverse events were rare, mild, and manageable (132, 144).						
	Limitations	Small samples, heterogeneous protocols, short follow-up, mostly subjective outcomes, and bias concerns.						
	Evidence Level	Emerging. Benefits are suggested, but certainty remains low/very low; larger sham-controlled RCTs are needed.						
Neurofeedback Biofeedback	Representative Study Design	Literature	Population	N	RCT	Placebo effect excluded	Objective biomarkers	Follow-up
		(136)	(a) diagnosed by DSM-4	82	No	No	Yes	No
		(137)	(a) diagnosed by DSM-5	17	Yes	No	Yes	Yes
		(138)	(c) cancer patients	28	Yes	No	Yes	No
	Main Clinical Findings & Safety	Possible sleep benefits in small studies, but surface neurofeedback did not outperform controls for insomnia/sleep quality (139). Stress biofeedback improved stress/anxiety/depression (140). No major safety concerns reported.						
	Limitations	Small samples, heterogeneous protocols, limited sham controls, mostly self-report, short/unclear follow-up, indirect populations.						
	Evidence Level	Emerging. Some supportive controlled/meta-analytic data, but insomnia-specific efficacy is inconsistent and limited by bias, heterogeneity, and weak long-term evidence.						
HVET	Representative Study Design	Literature	Population	N	RCT	Placebo effect excluded	Objective biomarkers	Follow-up
		(148)	(b) adults	100	No	No	No	No
		(149)	(b) adults	40	No	No	No	No
		(150)	(a) Chinese Guidelines for the Diagnosis and Treatment of Adult Insomnia, 2017 edition	88	Yes	No	No	No
	Main Clinical Findings & Safety	Studies suggest improved PSQI/sleep quality; one add-on RCT also reported fewer adverse events. No major safety issues reported.						
	Limitations	Small, short-term; limited randomization; no sham control; subjective outcomes only; no follow-up.						
	Evidence Level	Exploratory. Evidence is mainly small/limited clinical data without adequate controlled validation.						

However, a critical appraisal of the current literature reveals several nuanced methodological and mechanistic challenges. First, true double-blinding is notoriously difficult in electrical stimulation trials; the perceptible somatosensory effects (e.g., tingling) of active stimulation often compromise sham integrity, suggesting that some reported sleep improvements may be partially conflated with placebo or expectancy effects (130, 131, 134, 135). Second, the assumption of consistent central neuromodulation in tES may oversimplify the reality of inter-individual anatomical differences (e.g., skull thickness and cerebrospinal fluid volume), which can significantly alter transcranial current shunting and cortical dosing (130, 131). Furthermore, while taVNS currently presents the most coherent clinical evidence base among these modalities, the exact pathways translating vagal afferent signaling into sustained sleep continuity remain to be fully elucidated, and the durability of such effects after treatment cessation is largely uncharacterized (132, 142, 143). Meanwhile, HVET lacks rigorous sham-controlled validation and should remain clearly classified as exploratory (148, 149, 151).

Electrical stimulation therapies represent promising but still insufficiently validated adjuncts to behavioral and pharmacological treatments for insomnia. To advance the field, future trials must prioritize active-sham paradigms to confirm true neurobiological efficacy, alongside studies assessing long-term durability. Ultimately, integrating real-time physiological feedback to create personalized, closed-loop systems will be crucial for optimizing these therapies. In synthesis, tES and neurofeedback remain emerging approaches for cortical hyperarousal, taVNS is an emerging option for autonomic hyperarousal, and HVET remains exploratory.

## 8 Magnetic stimulation therapies

### 8.1 Conceptual framework and central magnetic neuromodulation

Repetitive transcranial magnetic stimulation (rTMS) is a non-invasive central neuromodulation technique that uses electromagnetic induction to modulate neuronal excitability without direct electrode contact (153). This “top-down” strategy is mechanistically aligned with insomnia features such as cortical hyperexcitation, impaired inhibition, and prefrontal-limbic network dysregulation (34, 154–156). rTMS can modulate cortical excitability bidirectionally in a frequency-dependent manner. Low-frequency stimulation (≤1Hz) is generally inhibitory, whereas higher-frequency stimulation (≥5Hz) enhances excitability (157). Low frequencies are normally used in treating insomnia (158–160). The goal is to attenuate hyperarousal and restore functional balance within sleep-relevant neural networks (155, 156, 159, 161). However, uncertainty remains regarding left versus right dorsolateral prefrontal cortex (DLPFC) stimulation, single-site versus network-guided targeting, and the influence of comorbid mood symptoms on target selection.

Neuroimaging has shown that rTMS can restore functional connectivity between the right DLPFC and limbic structures implicated in emotional regulation and arousal (156). Network-level analyses further reveal that rTMS normalizes small-world properties of brain networks (161), indicating improved efficiency and integration of information processing. Moreover, rTMS reconfigures dynamic connectivity across large-scale networks, exemplified by restored coupling between the default-mode and visual networks (159). On the molecular and cellular levels, rTMS has been shown to regulate neurotrophic factors, neurotransmitter systems, and inflammation. It may increase BDNF and GABA levels (162) while reducing pro-inflammatory cytokines (163), though the specific pathways and their causal links to symptom relief require further validation. Predictive models incorporating electroencephalographic coherence and network features have now been proposed to guide the development of individualized stimulation protocols (164).

A growing but heterogeneous body of clinical evidence suggests that rTMS may improve insomnia symptoms, particularly subjective sleep quality (158, 160, 165, 166). Pooled evidence from systematic studies and meta-analyses indicates that rTMS improves both subjective perceptions of sleep quality and objective polysomnographic measures, with 1Hz stimulation being the most commonly used (160), but repeated courses may be needed for maintenance (166). Beyond primary insomnia, rTMS may help improve sleep disturbances associated with neurological and neuropsychiatric conditions, including Parkinson’s disease, post-stroke insomnia, and neurodevelopmental disorders such as attention-deficit/hyperactivity disorder and autism spectrum disorder (167–169).

### 8.2 PMTS, PEMF, and other low-intensity magnetic interventions

Pulse magnetic therapy system (PMTS), pulsed electromagnetic field therapy (PEMF) and other lower-intensity magnetic treatments are now considered the most accessible alternatives. A multicenter, randomized, double-blind trial has reported significant reductions in insomnia severity with PMTS treatment (170). Other studies suggest potential benefits in specific contexts, but the evidence is weaker and more device-dependent than that supporting rTMS, and it is often less specific to insomnia (171, 172). The precise biophysical mechanisms underlying the effectiveness of low-intensity PMTS and PEMF remain unclear. They may involve modulating neuronal excitability or systemic autonomic tone. For now, those treatments are best viewed as adjunctive or maintenance candidates, pending larger standardized trials. A comparative breakdown of these magnetic and electromagnetic interventions is presented in **Table 5**.

**Table 5:** Magnetic stimulation therapies for insomnia.

Therapy	Evaluation item	Evaluation results							
rTMS	Representative Study Design	Literature	Population	N	RCT	Placebo effect excluded	Objective biomarkers	Follow-up
		(156)	(a) diagnosed by ICSD-3	44	Yes	No	Yes	No
		(158)	(a) diagnosed by DSM-5	49	Yes	Yes	Yes	No
		(159)	(a) diagnosed by DSM-5	26	No	No	Yes	No
		(165)	(a) diagnosed by ICSD-3	53	Yes	Yes	No	Yes
		(166)	(a) diagnosed by DSM-IV-TR, Chinese Classification of Mental Disorders and Diagnostic Criteria	70	Yes	No	Yes	Yes
	Main Clinical Findings & Safety	rTMS improves subjective insomnia/PSQI short-term; PSG results are mixed (160). Biomarker effects are possible but inconsistent (162, 163). Generally safe, mainly mild headache.						
	Limitations	Small samples, heterogeneous protocols, limited blinding/sham control, short follow-up, and weak objective validation.						
	Evidence Level	Moderate for subjective sleep improvement; emerging overall. PSQI benefits are supported, but certainty is limited by heterogeneity, bias risk, short follow-up, and weak objective validation. High-certainty evidence for routine stand-alone insomnia treatment is lacking.						
PMTS PEMF	Representative Study Design	Literature	Population	N	RCT	Placebo effect excluded	Objective biomarkers	Follow-up
		(170)	(a) diagnosed by DSM-5	153	Yes	Yes	No	Yes
		(171)	(c) nocturia patients	35	Yes	No	No	Yes
		(172)	(c) post-COVID-19 fatigue syndrome patients	20	Yes	Yes	No	Yes
	Main Clinical Findings & Safety	Improved sleep/insomnia scores and QoL; well tolerated, no serious AEs.						
	Limitations	Small samples, short follow-up, mostly subjective outcomes, heterogeneous protocols.						
	Evidence Level	Emerging. Controlled evidence suggests benefit, but limited by bias, size, and indirect populations.						

### 8.3 Summary

Magnetic stimulation, particularly rTMS, is receiving increasing attention as a treatment for insomnia (158, 160, 165, 166). Some rTMS network effects have been demonstrated in insomnia patients, whereas many molecular mechanisms and most PMTS/PEMF mechanisms are still inferred from related populations, device-specific studies, or preclinical evidence. The rTMS evidence base is relatively well developed (156, 159, 161–164), but it remains heterogeneous. Low-frequency prefrontal application has more often been linked to improved sleep outcomes, but the data are somewhat inconsistent (158, 160, 165, 166). In studies of rTMS for insomnia, stimulation frequencies typically range from 0.5 to 20 Hz, with low-frequency 1 Hz stimulation being the most frequently used (160); however, large-scale, multicenter, rigorously sham-controlled randomized trials remain scarce.

Meanwhile, lower-intensity modalities such as PMTS and PEMF may offer more accessible options (170), but their efficacy appears device-dependent. However, their specific biophysical mechanisms in relation to sleep regulation remain somewhat ambiguous compared to the clearer top-down cortical targeting of rTMS. Given that current efficacy reports appear to be highly device-dependent (170–172), their clinical positioning is tentatively viewed as adjunctive or for maintenance.

Future work across all magnetic modalities should prioritize parameter optimization and evaluation of long-term durability (166). Specifically, advancing neuroimaging/electrophysiology-guided targeting for rTMS and conducting independent, standardized replications for PMTS/PEMF devices will be essential to clarify their respective roles within multimodal or precision-oriented insomnia care. Overall, rTMS is an emerging-to-moderate adjunct for insomnia with prominent cortical or affective hyperarousal, whereas PMTS and PEMF remain emerging, device-dependent adjuncts.

## 9 Physical factor therapies, CBT-I, and medication compared

### 9.1 CBT-I and medication

CBT-I is widely recognized as a first-line treatment for insomnia because it is effective and the effects are durable (8, 30). However, real-world implementation faces substantial barriers, including a lack of trained therapists, its time-consuming protocols, and often poor adherence in routine clinical practice (30, 31). Importantly, CBT-I primarily targets cognitive-behavioral mechanisms, so it may not adequately address all of insomnia’s physiological dimensions—such as autonomic or circadian dysregulation—when used in isolation (36, 47).

Medication provides rapid symptomatic relief and remains widely used in clinical practice (8, 30). Nevertheless, long-term use risks tolerance, dependence, residual daytime sedation, and other adverse effects, especially among the elderly and those with some comorbidity (32, 33).

### 9.2 The role of physical factor therapies

Physical factor therapies directly target sleep’s neurobiological underpinnings, including cortical hyperexcitability, circadian rhythm misalignment, and autonomic imbalances (60, 86, 102, 131, 156). They should not be regarded as replacements for CBT-I, but may serve as mechanism-oriented adjunctive therapies in selected patients (129, 137, 173). By modulating physiological arousal and neural network dynamics, physical factor therapies may enhance responsiveness to CBT-I, reduce drug reliance, and provide viable alternatives for those intolerant of, or unresponsive to, other treatments (47, 133). In practice, their use is also shaped by implementation practicalities. Access to CBT-I is limited by a lack of therapists in many places, and adherence is demanding (30, 31). Medication is straightforward, but constrained by safety and long-term tolerance concerns (32, 33). For physical factor therapies, particularly device-based modalities, formal cost-effectiveness data remain limited, and their real-world value is likely to depend on sustained adherence and appropriate patient selection. In this context, physical factor therapies may function as adjunctive “enablers” that reduce physiological arousal or circadian instability and thereby support sustained relief.

### 9.3 Phenotype matching and precision sleep medicine

**Figure 2:**
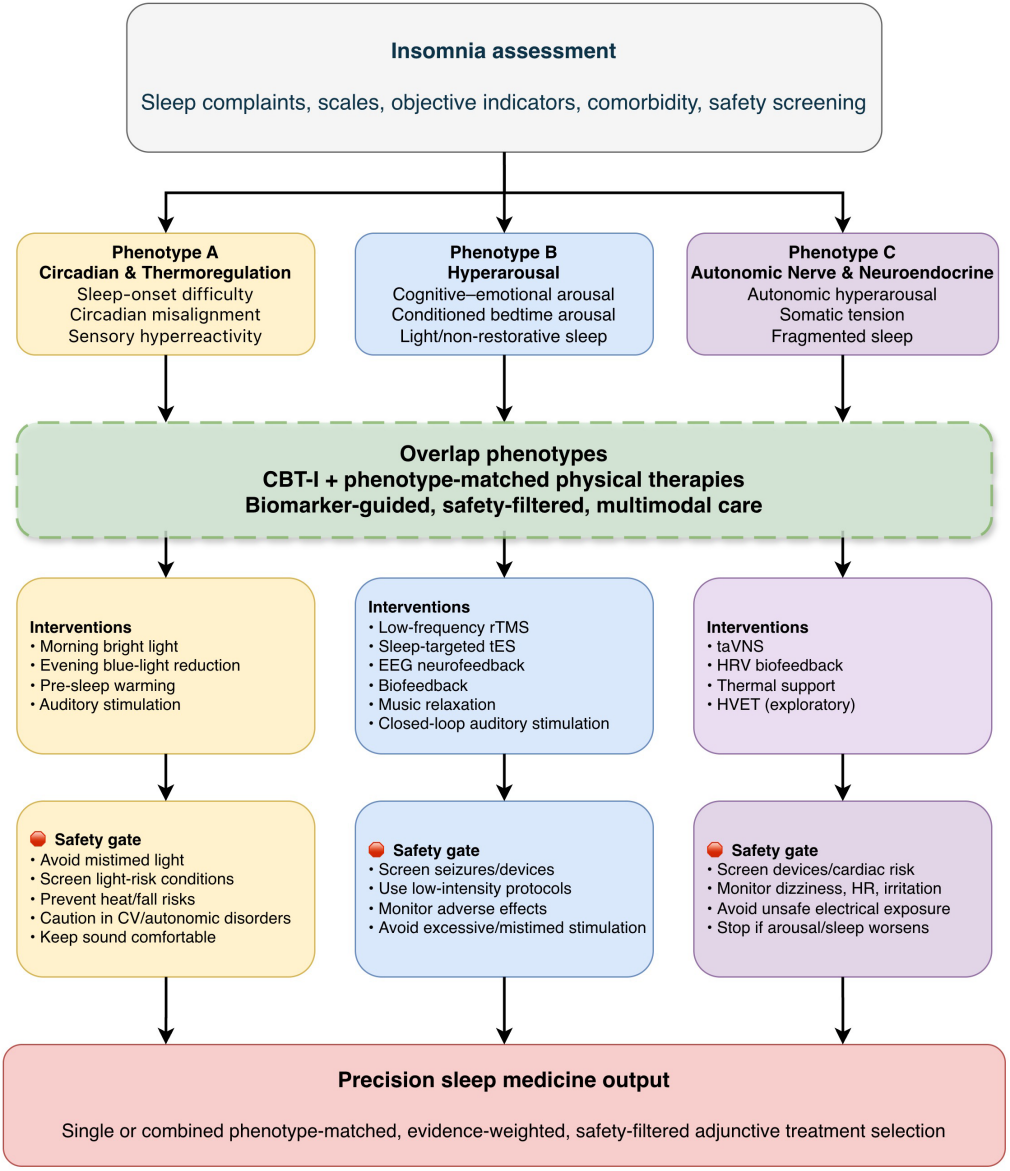
Proposed exploratory framework for phenotype-guided selection of physical factor therapies for insomnia. CBT-I, cognitive behavioral therapy for insomnia; rTMS, repetitive transcranial magnetic stimulation; tES, transcranial electrical stimulation; EEG, electroencephalography; taVNS, transcutaneous auricular vagus nerve stimulation; HRV, heart rate variability; HVET, high-voltage electrostatic therapy; CV, cardiovascular; HR, heart rate.

Insomnia’s heterogeneity calls for precision in its treatment. **Figure 2** illustrates a proposed conceptual framework for translating the mechanism-based taxonomy shown in **Figure 1** into phenotype-guided therapy selection in clinical practice. In such a framework, circadian and/or sensory dysregulation, and the various forms of hyperarousal are treated as dominant but potentially overlapping. This framework may help generate testable hypotheses for phenotype-matched, evidence-weighted, and safety-filtered adjunctive treatment selection.

Sleep-onset insomnia related to circadian delay may be particularly suitable for timed light therapy, while sleep-onset difficulties involving impaired heat dissipation may respond to thermal interventions such as pre-sleep passive body heating or distal warming (91, 129).

Those with pronounced cognitive or emotional hyperarousal may be considered potential candidates for central neuromodulation techniques, including transcranial magnetic or electrical stimulation, although phenotype-specific evidence remains limited (130, 158). Those with prominent autonomic dysregulation may be reasonable candidates for interventions such as taVNS (134), for which evidence supporting its use in insomnia is emerging. HVET remains exploratory and should not be considered a proven option at present (147–149, 151).

Conceptually, combining physical factor therapies with CBT-I may be advantageous, but direct clinical evidence remains limited. A physical factor therapy could reduce hyperarousal or circadian instability, and that could facilitate engagement with cognitive-behavioral treatment. However, direct evidence supporting the utility of such combinations remains limited (129, 137, 173). Such multimodal approaches could be particularly relevant for treatment-resistant insomnia (82, 174), where single-modality interventions frequently fail to achieve a satisfactory outcome.

## 10 Limitations and future directions

### 10.1 Limitations of the current evidence base

Interest in physical factor therapies is growing, but the evidence base remains limited by small samples, short interventions, overly brief follow-ups, possible bias, and inconsistent reporting (68, 92, 111, 132, 139, 160). Different study designs, stimulation parameters, outcome measures and control conditions hamper inter-study comparison and quantitative synthesis.

Another major limitation is the heavy reliance on subjective sleep scales (e.g., the PSQI and ISI). They are clinically relevant, but vulnerable to expectancy and context effects, especially in trials of physical factor therapies involving device-based delivery, where sham credibility and blinding are difficult to achieve and are not consistently assessed or reported. That weakens internal validity and inflates the risk of bias (90, 113, 134, 170). Inconsistent use of polysomnography (PSG) and actigraphy also complicates causal and mechanistic interpretation of the data reported (69, 115, 135, 158, 164).

Future research should more routinely incorporate objective sleep measures like polysomnography, actigraphy and relevant physiological biomarkers. And the credibility of sham treatment and blinding should be better reported. Co-primary endpoints that pair a patient-centered outcome meeting a predefined minimal clinically important difference (MCID) with at least one objective physiological marker should be used where feasible. That would enhance interpretability and assay sensitivity.

### 10.2 Challenges in standardization and individualized application

One difficulty in applying physical factor therapies in routine clinical practice lies in the lack of standard treatment protocols. Differences in stimulation intensity, timing, frequency and treatment duration hamper reproducibility and the development of clinical guidelines (68, 109, 132, 152, 160). At the same time, excessive standardization risks neglecting substantial individual differences in insomnia’s pathophysiology, including variations in cortical excitability, autonomic tone, and circadian phasing (44, 49, 106, 161).

Future research therefore needs to balance the harmonization of treatment protocols against adaptive personalization. Frameworks that define core parameter ranges while allowing data-driven individual adjustment may offer a pragmatic pathway toward scalable yet flexible clinical implementation.

### 10.3 Gaps in mechanistic understanding

Clinical improvements following physical factor therapy are often reported, but direct causal links remain incompletely established (51, 133, 162). Most investigators have inferred mechanisms indirectly; few have integrated longitudinal neurophysiological measurements with clinical outcomes (83, 143, 156). Tests of multimodal approaches combining electrophysiology with neuroimaging and supported by autonomic markers are urgently needed to delineate how changes in brain networks, oscillation dynamics and autonomic regulation translate into improved sleep continuity and quality. Establishing mechanistic biomarkers will also be essential for identifying responders and refining treatment selection.

### 10.4 Toward precision and closed-loop sleep medicine

Stratifying patients based on physiological and circadian profiles enables targeted interventions (17, 44, 129). Key biomarkers facilitating this include electroencephalography (EEG) coherence to predict neuromodulation outcomes (164), dim light melatonin onset (DLMO) to guide the timing of light-based circadian interventions (104, 106, 129), and heart rate variability (HRV) to track autonomic hyperarousal (48, 49).

Wearables and home monitoring enable continuous physiological tracking in real-life settings (49, 58, 130). Integrated with real-time signal processing, they drive adaptive closed-loop systems. In insomnia, EEG-guided auditory stimulation enhances slow-wave sleep (64, 69) and accelerates sleep onset (70), while physiological feedback optimizes efficacy and minimizes adverse effects (61, 65).

Advances in portable, home-based technologies support their long-term integration (88, 90, 118, 119, 130, 134, 138). However, clinical translation requires addressing accessibility and cost-effectiveness. Converting laboratory systems into affordable home devices could help these personalized treatments reduce the economic burden of insomnia (28, 29).

## 11 Conclusion

Insomnia involves interacting disturbances in cortical arousal, autonomic regulation, circadian timing, thermoregulation, and cognitive-emotional processing. Physical factor therapies offer a mechanistically plausible and rapidly evolving group of non-invasive adjunctive interventions that may target selected components of this pathophysiology. Current evidence suggests that phototherapy may be useful for circadian or delayed-phase phenotypes, thermotherapy for sleep-onset difficulties related to thermoregulatory impairment, rTMS for cortical hyperarousal, and taVNS for autonomic dysregulation, but these applications remain phenotype-specific and require further validation. Auditory stimulation, tES, neurofeedback, PMTS/PEMF, and other modalities remain promising but require more rigorous validation; HVET remains exploratory.

Overall, these therapies should be regarded as potential adjunctive approaches for selected insomnia presentations rather than established routine replacements for CBT-I or medication. The field now requires larger sham-controlled trials, standardized protocols, objective sleep and circadian outcomes, longer follow-up, adverse-event reporting, and phenotype-stratified designs. If validated, physical factor therapies may eventually contribute to personalized, mechanism-based insomnia management.
