# Supplementary material for: Physical factor therapies for insomnia: a narrative review of mechanisms, clinical evidence, and phenotype-guided applications
Source: Front Psychiatry. 2026 Jun 22;17:1835178. doi: 10.3389/fpsyt.2026.1835178 (PMC13333621; doi:10.3389/fpsyt.2026.1835178)
Supplement: Supplementary file 1 [file SupplementaryFile1.docx]

**Methods**

**Review Design**

This review was designed as a semi-structured narrative review, not as a systematic review or meta-analysis. The aim was to summarize mechanisms, clinical evidence, safety considerations, and potential phenotype-guided applications of non-invasive physical factor therapies for insomnia and insomnia-related sleep disturbance. Because the included literature varied substantially in intervention type, population, comparator, and outcome measures, no quantitative meta-analysis was performed.

**Search Strategy**

A literature search was conducted in PubMed/MEDLINE, Scopus, Web of Science Core Collection, J-STAGE, the Chinese Medical Journal Network, and CNKI. Google Scholar and manual screening of reference lists were used to identify additional relevant publications. The search covered publications from database inception to May 2026. Publications in English, Chinese, and Japanese were considered.

Search terms combined insomnia-related terms with intervention-specific terms. Insomnia-related terms included “insomnia”, “insomnia disorder”, “sleep disturbance”, “sleep quality”, “sleep onset latency”, “wake after sleep onset”, and “circadian rhythm sleep-wake disorder”. Intervention-related terms included “auditory stimulation”, “closed-loop auditory stimulation”, “white noise”, “pink noise”, “music therapy”, “bright light therapy”, “phototherapy”, “light therapy”, “blue light”, “thermotherapy”, “warming”, “footbath”, “bathing”, “sauna”, “transcranial electrical stimulation”, “tDCS”, “tACS”, “transcutaneous auricular vagus nerve stimulation”, “taVNS”, “neurofeedback”, “biofeedback”, “high-voltage electrostatic therapy”, “high-potential therapy”, “repetitive transcranial magnetic stimulation”, “rTMS”, “pulsed magnetic therapy”, “pulsed electromagnetic field”, and “PEMF”. Equivalent Chinese and Japanese terms were used when searching Chinese and Japanese databases.

**Eligibility Criteria**

Clinical efficacy appraisal was restricted to adult human studies. Eligible clinical evidence included randomized controlled trials, randomized crossover studies, controlled clinical studies, systematic reviews, and meta-analyses evaluating non-invasive device-based or sensory physical stimulation therapies in adults with diagnosed insomnia disorder, insomnia symptoms, or clinically relevant sleep disturbance. Studies involving medical, psychiatric, neurological, cancer-related, nocturia-related, post-COVID, or shift-work populations were included but were considered indirect evidence when formal insomnia disorder was not diagnosed. Mechanistic studies, animal studies, uncontrolled observational studies, and case series were not used as primary evidence for clinical efficacy, but were considered for mechanistic plausibility, feasibility, or safety discussion. Screening was performed by one author and verified by another. Disagreements or uncertainties were discussed with a senior author.

**Population Classification**

Evidence was categorized by population type to avoid overgeneralization across heterogeneous samples. Population categories were defined as follows:

(a) diagnosed insomnia disorder according to ICSD, DSM, or equivalent clinical criteria;

(b) insomnia symptoms or elevated insomnia scale scores without formal diagnostic confirmation;

(c) comorbid sleep disturbance in medical, psychiatric, neurological, cancer-related, nocturia-related, or post-COVID populations;

(d) circadian rhythm or shift-work-related sleep disturbance;

(e) mechanistic, experimental, healthy-volunteer, or preclinical evidence without direct clinical insomnia outcomes.

**Evidence Appraisal**

A GRADE-informed body-of-evidence framework was used to classify clinical efficacy evidence as exploratory, emerging, moderate, or high. This was not a formal study-by-study GRADE assessment. Ratings were based on study design, population relevance, risk of bias, consistency, precision, directness to insomnia disorder, adequacy of comparator or sham control, follow-up duration, and use of objective sleep outcomes.

Evidence levels were defined as follows. Exploratory evidence referred to mechanistic, preclinical, observational, uncontrolled, or small pilot evidence without adequate controlled clinical validation. Emerging evidence referred to at least one controlled clinical study suggesting potential benefit, but with important limitations such as small sample size, risk of bias, indirect population, inconsistent findings, or short follow-up. Moderate evidence referred to multiple controlled studies or systematic reviews showing broadly consistent benefit, while important limitations remained. High evidence referred to multiple well-conducted, adequately powered, sham-controlled randomized trials in insomnia disorder with consistent findings and sufficient follow-up. No modality was considered to have high-certainty evidence for routine stand-alone treatment of insomnia disorder.

Mechanistic plausibility was summarized separately and did not increase the clinical evidence rating unless supported by controlled clinical data in insomnia-relevant populations. Evidence from comorbid sleep disturbance, shift-work populations, healthy volunteers, or preclinical models was treated as indirect when drawing conclusions about adults with insomnia disorder.

**Supplementary Table S1. Search strategy and evidence classification summary**

| Item | Description |
| --- | --- |
| Review type | Semi-structured narrative review and evidence map |
| Databases | PubMed/MEDLINE, Scopus, Web of Science Core Collection, J-STAGE, Chinese Medical Journal Network, CNKI |
| Additional sources | Google Scholar, citation tracking, and references of relevant reviews and trials |
| Search period | Database inception to May 2026 |
| Language | In English, Chinese, and Japanese |
| Population | Adults with diagnosed insomnia disorder, insomnia symptoms, poor sleep quality, comorbid sleep disturbance, or circadian/shift-work sleep disturbance |
| Interventions | Auditory stimulation, phototherapy, thermotherapy, tES, taVNS, neurofeedback/biofeedback, HVET, rTMS, PMTS/PEMF |
| Study types prioritized | SR/MAs, RCTs, randomized crossover trials, controlled clinical studies, sham-controlled trials |
| Indirect evidence | Comorbid, neurological, cancer-related, nocturia-related, post-COVID, and shift-work studies were treated as indirect evidence when formal insomnia disorder was not diagnosed |
| Mechanistic evidence | Used only for biological plausibility, not as primary evidence for clinical efficacy |
| Screening | Performed by one author and verified by another |
| Formal PRISMA/RoB/GRADE | Not conducted; certainty descriptors were informed by GRADE domains |
| Evidence levels | Exploratory, emerging, moderate, high |
